# Seropositivity of Anti-*Toxoplasma gondii* Antibodies in Owners and Their Dogs Living on Island and Mainland Seashore Areas of Southern Brazil

**DOI:** 10.3390/tropicalmed7100252

**Published:** 2022-09-20

**Authors:** Aaronson Ramathan Freitas, Ruana Renostro Delai, Louise Bach Kmetiuk, Evelyn Cristine da Silva, Rafaella Martini, Ana Pérola Drulla Brandão, Rogério Giuffrida, Ivan Roque de Barros-Filho, Rodrigo Costa da Silva, Hélio Langoni, Fabiano Borges Figueiredo, Cláudia Turra Pimpão, Andrea Pires Dos Santos, Vamilton Alvares Santarém, Alexander Welker Biondo

**Affiliations:** 1 Department of Veterinary Medicine, Federal University of Paraná State, Curitiba 80035-050, PR, Brazil; 2 Laboratory of Cell Biology, Carlos Chagas Institute, Oswaldo Cruz Foundation, Curitiba 81310-020, PR, Brazil; 3 Department of Veterinary Hygiene and Public Health, São Paulo State University, Botucatu 18618-681, SP, Brazil; 4 Department of Preventive Medicine, University of São Paulo, São Paulo 05508-270, SP, Brazil; 5 Laboratory of Veterinary Parasitology, Veterinary Teaching Hospital, University of Western São Paulo, São Paulo 190019-70, SP, Brazil; 6 Department of Animal Science, School of Life Sciences, Pontifical Catholic University of Paraná, Curitiba 80230-130, PR, Brazil; 7 Department of Comparative Pathobiology, College of Veterinary Medicine, Purdue University, West Lafayette, IN 47907, USA

**Keywords:** Brazil, One Health, pets and human health, serosurvey, toxoplasmosis, zoonosis

## Abstract

Although toxoplasmosis has been considered among the most neglected zoonoses worldwide, no study has focused on the frequency and associated risk factors of owners and their dogs living on an island and mainland seashore areas. Accordingly, anti-*Toxoplasma gondii* IgG antibodies were screened by indirect fluorescent antibody test (IFAT) in owners and dogs from three oceanic islands and two nearby mainland harbor areas, with associated risk factors for toxoplasmosis assessed by univariable and multivariable logistic regression models. Overall, anti-*T. gondii* seropositivity was observed in 59/328 (18.0%) owners and 66/283 (23.3%) dogs, with no statistical difference between owners (*p* = 0.360) and dogs (*p* = 0.655) from islands and mainland areas. Consumption of local water springs (*p* = 0.016; OR = 2.11) was an associated risk factor for *T. gondii* seropositivity, and owners with the habit of spring water intake were twice more likely seropositive (*p* = 0.014; OR = 2.14). Presence of anti-*T. gondii* antibodies in dogs was associated with seropositive owners (*p* = 0.008; OR = 2.81), household consumption of beef meat (*p* = 0.042; OR = 1.7) and chicken (*p* = 0.026; OR = 2.9). Despite being lower than the worldwide prevalence, toxoplasmosis seropositivity in owners and their dogs in southern Brazil was influenced by the positive owner, water source, and meat consumption, and not by inhabiting islands or seashore mainland areas, presence of dogs, cats, or both. In addition, drinking water quality should always be considered a critical risk factor for toxoplasmosis on islands.

## 1. Introduction

Toxoplasmosis has been described as a worldwide distributed zoonosis caused by *Toxoplasma gondii*, a protozoan having cats and other Felidae as definitive hosts, which shed oocysts into feces to infect a variety of intermediate hosts, including dogs and human beings [1]. As shed *T. gondii* oocysts may be environmentally viable and retain longtime infectivity, particularly under optimal weather conditions, a One Health approach should be applied to concomitantly assess the human, animal, and environmental role on pathogen transmission [2].

Previous studies have already shown *T. gondii* overspreading in the Brazilian oceanic island of Fernando de Noronha, a major national preservation park and touristic destination 545 km far (340 miles) from the Brazilian mainland seashore, with a human seroprevalence of 172/341 (50.4%) inhabitants, along with high prevalence of 248/348 (71.3%) pet and 135/247 (54.7%) feral cat populations [3,4]. In this confined scenario of islands and seashore areas, a potential exacerbated transmission may occur due to favorable climate and daily exposure, as previously shown in the same areas studied herein for toxocariasis [5].

Dogs have been considered sentinels for human toxoplasmosis due to domiciliation and household sharing, mostly exposed the same homemade food and water sources [6,7,8]. Although seroprevalence studies of dog toxoplasmosis in Brazil have ranged from 7.95% [9] to 40.0% [10], Fernando de Noronha Island has shown surprising seropositivity of 156/320 (48.8%) dogs [4]. In addition, human toxoplasmosis has been considered one of the six neglected parasitic diseases, along with Chagas, cyclosporiasis, cysticercosis, toxocariasis and trichomoniasis, demanding worldwide public health action [11].

Despite the consideration of toxoplasmosis as one of the most neglected zoonoses worldwide, no study to date has focused on the frequency and associated risk factors of owners and their dogs living on island and mainland seashore areas.

## 2. Materials and Methods

### 2.1. Study Design

The current study was designed as a cross-sectional seroepidemiological approach to owner and dog populations inhabiting oceanic islands and seashore mainland areas in the Paraná State, southern Brazil, from July 2019 to February 2020. The study herein was approved by the Ethics Committee of Animal Use at the Federal University of Parana (protocol 036/2021) and the Ethics Committee in Human Health at the Brazilian Ministry of Health (protocol 46994521.0.0000.0102).

### 2.2. Local of Study

The serological survey was performed on three oceanic islands, Superagui Island, Ilha do Mel Island, and Peças Island, and two seashore mainland areas of the cities of Guaraqueçaba and Pontal do Paraná, located in the Paraná State, southern Brazil. The three Islands were environmentally protected units at the time, with Ilha do Mel Island State Park and Superagui Island National Park located within the largest unfragmented area of the Brazilian Atlantic Forest biome [12].

The largest site, Ilha do Mel Island (25°32′32.16″ S and 48°18′15.67″ W), comprises two major uninhabited conservation parks (Ecological Station and State Park), protected by environmental laws and representing 93.4% of the total area of 2762.0 hectares [12]. Despite a local community of traditional culture with only 1100 permanent inhabitants, the island was ranked as the second biggest state touristic destination with 200,000 visitors annually. Currently distributed in five villages, local residents rely primarily on tourism for living [13].

The Superagui Island (25°27′25.31″ S and 48°14′46.05″ W) holds the Superagui National Park, a conservation unit adjacent to local traditional artisanal fisherman communities. The sampling point of Barra do Superagui with approximately 700 inhabitants and 350 dogs, was the major community at the time [14]. The smallest site, Peças Island (25°27′22″ S and 48°20′07″ W) were classified as fully environmentally protected area by the Brazilian government, particularly due to a dolphin nursery area [14]. Historically established during slavery, the Peças Island was inhabited by around 350 persons living on artisanal fishing and tourism at the time.

**Figure 1:**
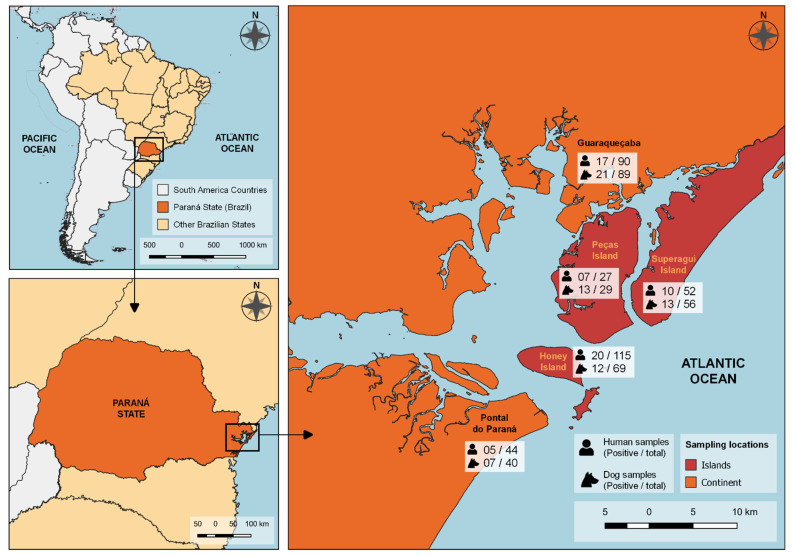
Sampling locations and frequency of anti-*T. gondii* antibodies in humans and their dogs from island and seashore mainland areas of southern Brazil.

In addition, the touristic and commercial harbors of Pontal do Paraná (25°40′26″ S and 48°30′39″ W) and Guaraqueçaba (25°18′25″ S and 48°19′44″ W) cities, two of the nearest seashore areas located at the northern Parana State coastal region, were sampled for mainland comparison (Figure 1) [13,14].

### 2.3. Blood Sample Collection

Populations inhabiting oceanic islands and seashore mainland areas were chosen by convenience, as owners were informed about the human and dog volunteer samplings by city officials when bringing their dogs for a series of free-of-charge campaigns of physical examination followed by high-quality high-volume spaying/neutering. Volunteer owners were then individually sampled by cephalic puncture after signed consent and completion of an epidemiological questionnaire, conducted by certified physicians and nurses at the local Basic Health Unit. Dogs were sampled by jugular puncture after signed owner consent and con-ducted by certified veterinarians. All samples were then placed in serum-separating tubes, centrifuged at 1500 RPM for five minutes, serum separated and stored at −20 °C until processing.

### 2.4. Serological Testing

Owner and dog serum samples were assayed for specific IgG antibodies against *T. gondii* by Indirect Fluorescent Antibody Test (IFAT) in five serial dilutions of phosphate-buffered saline pH 7.2 (PBS: 8.2 g NaCl: 1.9 g Na_2_HPO_4_·7H_2_O and 0.3 g NaH_2_PO_4_·H_2_O) solution, including 1:16, 1:64, 1:256, 1:1024 and 1:4096. Immunofluorescence slides were priorly sensitized with 0.1% formaldehyde to inactivated *T. gondii* tachyzoites (RH strain), and then applied for fluorescence using a commercial anti-IgG (human or dog) specific antibody conjugated with fluorescein isothiocyanate (Bethyl-Montgomery, TX, USA). Readings were performed by a certified researcher using an immunofluorescence microscope.

Seropositive samples were confirmed with antibody titers ≥ 16 for *T. gondii*, as the established cutoff point, and final serum titers were determined by the highest dilution with ≥50% tachyzoites still showing fluorescence. Positive and negative human and dog controls were added to each slide to ensure quality control in all readings.

The Indirect Fluorescent Antibody Test (RIFI) was performed in this study, as it is recommended as the gold standard, described with greater specificity for the diagnosis of *T. gondii* [15,16] and comparable sensitivity to the Enzyme-linked Immunosorbent Assay (ELISA) and Modified Agglutination Test (MAT) [16,17,18]. Serum samples from the owner and dog were screened for specific IgG antibodies against *T. gondii* by the Indirect Fluorescent Antibody Test (IFAT). Serum samples from the owner and dog were screened for specific IgG antibodies against *T. gondii* by the Indirect Fluorescent Antibody Test (IFAT) in five serial dilutions of phosphate-buffered saline pH 7.2 (PBS: 8.2 g NaCl: 1.9 g Na_2_HPO_4_·7H_2_O and 0.3 g NaH_2_PO_4_·H_2_O) solution, including 1:16, 1:64, 1:256, 1:1024 and 1:4096, according to other studies that advocated this diagnostic method [19,20,21,22].

Immunofluorescence slides were previously sensitized with 0.1% formaldehyde to inactivated tachyzoites of *T. gondii* (RH strain), and then applied for fluorescence using a commercial anti-IgG specific antibody (human or canine) conjugated to fluorescein isothiocyanate (Bethyl Laboratories, Inc., Montgomery, TX, USA). Readings were taken by a certified researcher using an immunofluorescence microscope.

### 2.5. Epidemiological Data Collection

Human-associated risk factor analyses were based on an epidemiological questionnaire assessing potential exposure to *T. gondii*, which included owner age, gender, education level, household income and location, animal (dog or cat) ownership, drinking water source, food washing before eating, other raw or undercooked meat consumption.

Dog-associated risk factors analyses were also based on epidemiological questionnaire data, including residence location, sex, cohoused with other dogs or cats, daily diet, drinking water source, raw meat intake, beach access and hunting habits.

### 2.6. Statistical Analysis

A data set was constructed according to place of sampling (islands versus mainland areas) with the stratified outcome, where data with any inconsistency type was discharged after analysis from the data set. Seropositivity to *T. gondii* and potential associated risk factors were tested by variable categorization. Questionnaire responses such as “unknown” or “not informed” were considered as lost information and removed from analyses when of insignificant impact (<10% of total). Variable was disposed in tables of double entry after categorization and tested for associations with anti-*T. gondii* seropositivity by Pearson’s chi-squared test. When one or more table spots presented expected values lower than 5, Fisher’s exact test was applied as an alternative

Variables showing statistical significance equal to or lower than 0.2 were submitted to the multivariate logistic regression model, using the stepwise backward-type selection method as a source for the best estimative of Akaike information criteria. Likelihood reasons per interval were projected in both univariate and multivariate models at 95% confidence (odds ratio), with all analyses considering a 5% significance level and conducted using the R package [23,24].

In the logistic regression analysis, the final solution of backward stepwise approach was determined after one step, removing one (consumption of water consumption) out of the five seropositivity predictors including gender, water consumption, spring water consumption, and wash fruits/vegetables; only using water for washing fruits and vegetable for owners. In dogs, final solution was determined after two steps, with deletion of two (ingestion of cattle beef and ingestion of fish) out of five predictors (seropositive owner; home food diet; cattle, chicken, or fish meat).

## 3. Results

Antibodies to *T. gondii* were detected in 59/328 (18.0%; 95% CI: 14.1–22.4%) owners (Figure 1). Despite a higher seropositivity on islands with 37/194 (19.1%; 95% CI: 14.2–25.2%) seropositive owners when compared to 22/134 (16.4%; 95% CI: 11.1–23.6%) positive owners on seashore mainland areas, no statistical difference was observed (*p* = 0.562; OR = 0.8).

According to the univariate analysis, the consumption of treated water (*p* = 0.047; OR = 0.5) was as a protective factor, while water from local springs (*p* = 0.016; OR = 2.1) was a risk factor for *T. gondii* seropositivity. By the logistic regression, only the consumption of spring water (*p* = 0.027; OR = 2.0) was significantly associated with *T. gondii* seropositivity (Table 1).

Antibodies to *T. gondii* were detected in 66/283 (23.3%; 95% CI: 18.8–28.6%) dogs (Figure 1). Despite a slightly higher seropositivity in dogs on seashore mainland with 38/154 (24.7%; 95% CI: 18.5–32.1%) when compared to 28/129 (21.7%; 95% CI: 15.5–29.6%) on island dogs, no statistical difference was observed (*p* = 0.655).

Logistic regression revealed the consumption of chicken meat (*p* = 0.028; OR = 2.9) and having a seropositive owner as risk factor for *T. gondii* seropositivity in dogs (Table 2). Evaluation of breed, age and dog hunting habits was impaired due to lack of precision on information given by dog owners, representing more than 10% of lost information. Thus, the variables were removed from analyses.

## 4. Discussion

To the authors’ knowledge, this was the first concomitant study on the occurrence of *T. gondii* antibodies in owners and their dogs living on islands and seashore mainland areas. The overall owner seropositivity in this study of the 18.0% was lower than most previous serosurveys, including the 49.8% in San Carlos Island, Venezuela [25] and all other Brazilian studies, with the 68.7% in the Pará State, northern Brazil [26], the 50.4% in the Fernando de Noronha Island, far northeastern Brazil [3], the 41.5% [27] and the 36.8% [9] in the same Parana State, southern Brazil, and average of the 32.7% in southern and central-western Brazil [28]. Seropositivity in the present study was higher than the 12.4% in Panama [29] and the 5.1% in provinces of China [30].

As previously shown in a nationwide USA survey, risk for IgG seropositivity increased with age and was higher in males, in persons living below the poverty level, and in persons with less than high school education [31]. Despite such results may reflect differences in cultural, socio-economic, sanitary, and religious characteristics, along with presence of domestic and wild animals, water source, animal and human health, seropositivity in this study was not statistically influenced by gender, age, educational level or family income. Besides age and educational level association to seropositivity, Fernando de Noronha Island has also presented significantly higher human and dog toxoplasmosis seropositivity than the present study, possibly due to a higher density of domestic cats as definitive protozoan hosts [3]. Although feral cat control programs have been conducted since 2007 in Fernando de Noronha, free-range and feral populations were estimated in a total of 1287 cats in 2017 [32]. In addition, climate differences between the northeastern Fernando de Noronha and the southeastern islands of the present study may have impacted on environmental persistence and spreading of *T. gondii*. While Fernando de Noronha presents tropical climate with dry (August–January) and rainy (February–July) seasons, with an average annual temperature of 26–27 °C and average rainfall of 1400 mm/year, the islands in this study present a super-humid tropical-subtropical climate with no dry season, average temperature of 22 °C and average rainfall of 2500 mm/year [33]. Although a previous study has shown no evident infectivity loss of *T. gondii* oocysts maintained between 10 and 25 °C for 200 days in water [34], lower rainfall and mild dry season may provide better opportunities for environmental persistence and transmission. Thus, further studies should be conducted to better understand whether human and dog toxoplasmosis in seashore and island areas may be associated with local pet and feral cat populations, rainfall and/or rainy/dry season interchange, household distance from seashore, touristic visitation, professional exposure to sand (boatmen, waiters and other beach workers) and beach walking frequency.

As previously established, the main transmission routes of toxoplasmosis may include tissue cyst ingestion in raw or undercooked meat of infected animals, ingestion of fresh vegetables or water contaminated with *T. gondii* oocysts from cat feces [35,36]. In this study, the human water source was the only variable showing significant differences, with spring water presenting twice more likelihood of *T. gondii* IgG antibodies. In addition, piped water has been more likely contaminated with oocysts than river and roof water, compared to other drinking-water sources [29]. Water springs may also be contaminated by *T. gondii* feces from wild felids reservoirs [37]. As human anti-*T. gondii* antibodies were also associated with drinking rain or well water (OR = 2.4) in the Fernando de Noronha Island [3], drinking water quality should be always considered a critical risk factor for toxoplasmosis on islands.

Rainy seasons may also facilitate the dispersion of oocysts in the environment, increasing exposure to *T. gondii* infection, even those not owning cat at home, but drinking water from water springs with felid access as may occur in this study [3]. Waterborne outbreaks of toxoplasmosis have been frequently associated with inappropriate home-filtered or unfiltered water consumption in Brazil [38]. In a previous study, unfiltered municipally treated water was epidemiologically related with 155 cases of toxoplasmosis in Southern Brazil in 2001 [39]. In another study, consumption of home-filtered water by faucet-mount filtered was suggested as risk factor for toxoplasmosis outbreak occurred in Northeastern Brazil in 2006, probably associated with one step of filtration occurs when the tap is unlocked [40]. In a recent study of potential source of 809 toxoplasmosis cases in Southern Brazil, bioassay with mice and piglets suggested *T. gondii* contamination in public treated water [41]. In urban areas, *T. gondii* oocysts may be resistant to usual water treatment with chlorinated products and decantation, probably associated with inner layers of the oocyst and sporocyst walls, as water supply used in these areas may usually come from distant unprotected areas and lead to human outbreaks by oocyst contamination of drinking water [42,43]. In addition, a recent systematic revew has shown that only radiation and pressure were effective in destroying oocysts, while disinfectants in water has insufficiently or no effect on *T. gondii* oocysts [44].

No association was observed in the present study when comparing the consumption of raw meat and seropositivity, which may suggest that animal protein consumption was mostly based on intake of cooked or non-contaminated fish/seafood, which may have reduced the likelihood of ingesting contaminated chicken or beef meat. Thus, although consumption of local spring water was a risk factor in the present study, the relatively lower seropositivity of *T. gondii* in human beings when compared to other studies in Brazil may be consequence of dietary habits, based on cooked and/or non-contaminated protein source (mainly fish/seafood), as chicken meat consumption and having a seropositive owner were risk factors for dog seropositivity, corroborating with such hypothesis. Not surprisingly, our research group has recently shown that dietary habits of homeless persons and persons with animal hoarding behavior may also impact in lower seropositivity than the general population, mostly due to low intake of raw or uncooked meat and fresh vegetables [9,45].

Nonetheless, the study has shown the 39.0% of seropositive individuals declaring consumption of chicken and/or beef meat. No study to date was conducted with domestic animals, such as cattle and chicken, in island and seashore mainland of Parana State. A study with 386 serum samples of free-range chickens of northwest Parana has shown 102/386 (26.4%) seropositive for *T. gondii* by IFA, 64/386 (16.6%) by MAT and 47/386 (12.2%) in both tests [46]. In addition, samples of 38/119 (31.9%) seropositive free-range chickens used for isolation were considered positives in mouse bioassay, with high diversity of isolates (*n* = 18) [46]. A recent review has shown that cattle *T. gondii* seroprevalence ranged from 25.8 to 48.5% in Parana State [47].

Although 124/327 (37.9%) owners declared having cat at home, no risk association was observed on seropositivity to *T. gondii* antibodies. A previous study in Northern Brazil has shown that, despite no statistical significance observed for water source or water treatment, having a cat represented 1.95-fold more chance to be seropositive [26]. Dogs have also been proposed as toxoplasmosis sentinels for human infection, mostly due to sharing inhouse spaces and related exposure to food and water sources [6,7,8]. The overall seropositivity in dogs in this study (23.3%) was lower than the 40.8% in Bahia State, northeastern Brazil [48], 40.0% [10] and the 25.4% in Sao Paulo State, southeastern Brazil [8], the 36.5% in Bahia State, northeastern Brazil [49], the 34% in Rio de Janeiro State, southeastern Brazil [50], and the 32.7% in southern and central-western Brazil [28]; only higher than the 16.3% [27] and the 7.95% in the same Parana State, southern Brazil [9]. In addition, the observed seropositivity herein was lower than the 72.7% in Cuba [7] and higher than the 18.6% [51] and the 21.6% in China [52].

In the present study, dog seropositivity has not been associated with cats living in the same household. Still controversial, contact with cats may [53] or may not be considered a significant risk factor for dogs [9,28,53]. However, dogs with seropositive owners in this study have shown 2.81-fold more likelihood of seropositivity than those with seronegative owners (*p* = 0.008), which may be related to owner-dog sharing of food and water intake, emphasizing the One Health nature of toxoplasmosis. Although dogs eating homemade food were more likely seropositive in a previous study, varying from 1.70 to 7.00-fold in Sao Paulo [8], such risk was not be present in another report [9]. Despite dog meat consumption may vary as *T. gondii* infection source and raw rat meat has previously increased seropositivity by 5.19-fold [28], no raw meat was a risk factor in this study, exception for chicken meat, probably uncooked, increasing serology by 2.90-fold. Intervention actions were indicated to local health services, including sanitary measures on appropriate food cooking and water source, which may control and prevent future infections [8].

Although owned dogs cohoused to cats were not at higher risk, cats have been well established to play crucial role on indirect epidemiologic chain of *T. gondii* cycle by shedding oocysts into their feces, contaminating soil and water sources [25]. Despite cat presence in islands may increase human and dog seropositivity, and dogs may serve as important sentinels for human exposure, cat contact or presence at home were not associated with infection in mainland urban settings [3,27].

As limitations, comparisons of *T. gondii* transmission between owners and their dogs living on islands and seashore mainland areas may be impaired due to household changing, since persons may have been born or previously lived in islands and moved to continent, or vice versa, or may work in daily boat transportation or commercial trading, with frequent mainland-island movement. In addition, the present study has been limited to investigate only *T. gondii* antibodies in owned dogs, not assessing unowned dogs and pet or feral cats, which may play a role in toxoplasmosis transmission due to free outdoors access, particularly nearby spring water source. In addition, the variables age, dog hunting behavior and dog breed were analysed only in the univariated analyses (Table S1). Data concerning age and dog hunting behavior showed expressive lost information (18.4 and 11.3%, respectively). Increase of risk for seropositivy was observed in dogs aging more than one year, but only one dog younger than a year was positive. Consequently, the range of CI 95% for the Odds Ratio was extremely large (1 to 8 years-old: OR = 8.79; CI 95% = 1.81–212, older than 8 years: OR = 16.4; CI 95% = 2.68–433) leading to a potential imprecision of results. Despite dog breed showed only 9/283 (3.2%) dogs with missing information, imprecision of answers given by owners made this variable not fitted for the final model of logistic regression.

Finally, educative programs should be established to reduce *Toxoplasma gondii* risk of exposure among travelers, sightseers and residents in endemic areas, particularly by avoiding springer water consumption [54].

## 5. Conclusions

The present study is the first concurrent report of anti-*T. gondii* in owners and correspondent dogs of islands and seashore mainland areas. Although different risk factors may interfere on both human and animal infection occurrence, individually or together, the present study has shown different associated risk factors for owners and their dogs. Human being *T. gondii* exposure has been associated with spring water consumption, and dog exposure was associated with seropositive owner, household consumption of beef meat and chicken. Dogs of seropositive owners were more likely seropositive than those of seronegative owners, probably related to owner-dog sharing of food and water, emphasizing the One Health nature of toxoplasmosis. In addition, drinking water quality should be always considered a critical risk factor for toxoplasmosis on islands.

## Figures and Tables

**Table 1:** Associated risk factors of anti-*Toxoplasma gondii* antibodies detected by IFAT test in humans from island and seashore mainland areas of southern Brazil (*n* = 328).

Variable			Bivariate Analysis		Multivariate Analysis	
	Anti-*T. gondii* Antibodies		OR (95% IC)	*p* Value	OR (95% IC)	*p* Value
	Seropositive No. (%)	Seronegative No. (%)				
	59/328 (18.0)	269/328 (82.0)				
**Household Location**				0.562		
Seashore mainland	22 (37.3)	112 (41.6)	Ref			
Island	37 (62.7)	157 (58.4)	0.8 (0.47–1.49)			
**Gender**				0.098		
Female	31 (52.5)	175 (65.1)	Ref		Ref	
Male	28 (47.5)	94 (34.9)	1.7 (0.94–2.98)		1.6 (0.87–2.85)	0.149
**Age (Years Old)**				0.322		
>18	55 (93.2)	258 (95.9)	Ref			
<18	4 (6.8)	11 (4.1)	0.6 (0.19–0.20)			
**Education Level**				0.682		
>Elementary school	37 (62.7)	179 (66.5)	Ref			
≤Elementary school	22 (37.3)	90 (33.5)	1.2 (0.65–2.12)			
**Income**				0.627		
>1 minimum wage	40 (70.2)	165 (65.7)	Ref			
≤1 minimum wage	17 (29.8)	86 (34.3)	0.8 (0.43–1.51)			
**Dog Owner**				0.856		
No	7 (11.9)	27 (10.0)	Ref			
Yes	52 (88.1)	242 (90.0)	0.8 (0.35–2.15)			
**Cat Owner**				0.579		
No	39 (66.1)	164 (61.2)	Ref			
Yes	20 (33.9)	104 (38.8)	0.8 (0.44–1.46)			
**Water Consumption**						
Treated water				0.047 *		
No	27 (45.8)	84 (31.2)	Ref			
Yes	32 (54.2)	185 (68.8)	0.5 (0.30–0.96)			
**Well Water**				1		
No	52 (88.1)	236 (87.7)	Ref			
Yes	7 (11.9)	33 (12.3)	1.0 (0.38–2.23)			
**Spring Water**				0.016		
No	33 (55.9)	196 (72.9)	Ref		Ref	
Yes	26 (44.1)	73 (27.1)	2.1 (1.17–3.78)		2.0 (1.08–3.57)	0.027
**Wash Fruits/Vegetables**						
Before consumption				0.118		
No	4 (6.8)	7 (2.6)	Ref		Ref	
Yes	55 (93.2)	260 (97.4)	0.4 (0.10–1.49)		0.3 (0.06–1.11)	0.054
**Only Water**				0.177		
No	14 (23.7)	90 (33.8)	Ref		Ref	
Yes	45 (76.3)	176 (66.2)	1.6 (0.87–3.24)		1.8 (0.87–4.0)	0.132
**Water and Vinegar**				0.546		
No	48 (81.4)	204 (76.7)	Ref			
Yes	11 (18.6)	62 (23.3)	0.8 (0.35–1.51)			
**Water with Sodium Hypochlorite**				0.297		
No	56 (94.9)	238 (89.5)	Ref			
Yes	3 (5.08)	28 (10.5)	0.5 (0.11–1.42)			
**Consumption of Raw or Undercooked Meat**				0.3		
No	36 (61.0)	185 (69.0)	Ref			
Yes	23 (39.0)	83 (31.0)	1.4 (0.78–2.55)			
**Cattle**				0.276		
No	38 (64.4)	193 (72.6)	Ref			
Yes	21 (35.6)	73 (27.4)	1.5 (0.79–2.65)			
**Pig**				1		
No	56 (94.9)	249 (93.6)	Ref			
Yes	3 (5.1)	17 (6.4)	0.8 (0.18–2.57)			
**Chicken**				0.746		
No	57 (96.6)	252 (94.7)	Ref			
Yes	2 (3.4)	14 (5.3)	0.7 (0.09–2.53)			
**Fish**				0.924		
No	49 (83.1)	225 (84.6)	Ref			
Yes	10 (16.9)	41 (15.4)	1.1 (0.50–2.34)			

* Variable fitted the criterion for logistic regression (*p* < 0.2) but was not selected by the backward stepwise logistic regression model (R package).

**Table 2:** Associated risk factors of anti-*Toxoplasma gondii* antibodies detected by IFAT test in dogs from island and seashore mainland areas of southern Brazil (*n* = 283).

Variable			Bivariate Analysis		Multivariate Analysis	
	Anti-*T. gondii* Antibodies		OR (95% CI)	*p* Value	OR (95% CI)	*p* Value
	Seropositive (%)	Seronegative (%)				
	66/283 (23.3)	217/283 (76.7)				
**Household Location**				0.655		
Seashore mainland	38 (57.6)	116 (53.5)	Ref			
Island	28 (42.4)	101 (46.5)	0.9 (0.48–1.48)			
**Sex**				1		
Female	32 (48.5)	106 (48.8)	Ref			
Male	34 (51.5)	111 (51.2)	1.0 (0.58–1.77)			
**Cohabitate with Another Dog**				0.485		
No	58 (87.9)	199 (91.7)	Ref			
Yes	8 (12.1)	18 (8.29)	1.5 (0.60–3.64)			
**Dog and Cat Cohabitate**				1		
No	64 (97.0)	211 (97.2%)	Ref			
Yes	2 (3.03)	6 (2.76)	1.2 (0.15–5.33)			
**Seropositive Owner**				0.016		
No	52 (78.8)	197 (90.8)	Ref		Ref.	
Yes	14 (21.2)	20 (9.22)	2.7 (1.23–5.60)		2.8 (1.30–6.0)	0.008
**Dog Diet**						
**Dry Food**				0.532		
No	2 (3.03)	12 (5.56)	Ref.			
Yes	64 (97.0)	204 (94.4)	1.8 (0.46–12.7)			
**Home Cooked**				0.054		
No	19 (28.8)	93 (43.1)	Ref		Ref	
Yes	47 (71.2)	123 (56.9)	1.9 (1.03–3.45)		1.7 (0.93–3.20)	0.089
**Meat Consumption**						
**Cattle**				0.042 *		
No	50 (75.8)	189 (87.1)	Ref			
Yes	16 (24.2)	28 (12.9)	2.2(1.06–4.28)			
**Pig**				0.22		
No	62 (93.9)	212 (97.7)	Ref			
Yes	4 (6.06)	5 (2.30)	2.7(0.63–11.1)			
**Chicken**				0.026		
No	57 (86.4)	206 (94.9)	Ref		Ref	
Yes	9 (13.6)	11 (5.07)	3.0 (1.12–7.56)		2.9 (1.10–7.51)	0.028
**Fish**				0.101 *		
No	60 (90.9)	209 (96.3)	Ref			
Yes	6 (9.09)	8 (3.69)	2.6 (0.81–7.97)			
**Water Consumption**						
**Tap Water**				0.243		
No	29 (43.9)	76 (35.0)	Ref			
Yes	37 (56.1)	141 (65.0)	0.7 (0.39–1.21)			
**Well Water**				0.923		
No	59 (89.4)	197 (90.8)	Ref			
Yes	7 (10.6)	20 (9.22)	1.2 (0.44–2.84)			
**Beach Access**				0.673		
No	23 (34.8)	84 (38.7)	Ref			
Yes	43 (65.2)	133 (61.3)	1.2 (0.67–2.12)			

* Variables fitting the criterion for logistic regression (*p* < 0.2) but no selected by the backward stepwise logistic regression model (R package).

## Data Availability

Not applicable.

## Supplementary Materials

The following supporting information can be downloaded at: https://www.mdpi.com/article/10.3390/tropicalmed7100252/s1, Table S1: Univariate analysis for accessing bred, age and hunting behavior as risk factor for anti-Toxoplasma gondii antibodies detected by IFAT test in dogs from island and seashore mainland areas of southern Brazil (*N* = 283). The variables were analyzed separately due to the number of missing (age and hunting behavior: lost of information higher than 10%) or imprecision of data regarding bred provided by dog’s owners.
