# Supplementary material for: Seropositivity of Anti-Toxoplasma gondii Antibodies in Owners and Their Dogs Living on Island and Mainland Seashore Areas of Southern Brazil
Source: Trop Med Infect Dis. 2022 Sep 20;7(10):252. doi: 10.3390/tropicalmed7100252 (PMC9611663; doi:10.3390/tropicalmed7100252)
Supplement: Supplementary file 1 [file tropicalmed-07-00252-s001.zip › tropicalmed-1826201-supplementary.pdf]

Tropical Medicine and Infectious Disease

Article

Seropositivity of Anti-*Toxoplasma gondii* Antibodies in Owners and Their Dogs Living on Island and Mainland Seashore Areas of Southern Brazil

Aaronson Ramathan Freitas [1], Ruana Renostro Delai [1], Louise Bach Kmetiuk [2], Evelyn Cristine da Silva [3], Rafaella Martini [1], Ana Pérola Drulla Brandão [4], Rogério Giuffrida [5], Ivan Roque de Barros-Filho [1], Rodrigo Costa da Silva [5], Hélio Langoni [3], Fabiano Borges Figueiredo [2], Cláudia Turra Pimpão [6], Andrea Pires Dos Santos [7], Vamilton Alvares Santarém [5] and Alexander Welker Biondo [1,7,*]

[1] Department of Veterinary Medicine, Federal University of Paraná State, Curitiba 80035-050, PR, Brazil
[2] Laboratory of Cell Biology, Carlos Chagas Institute, Oswaldo Cruz Foundation, Curitiba 81310-020, PR, Brazil
[3] Department of Veterinary Hygiene and Public Health, São Paulo State University, Botucatu 18618-681, SP, Brazil
[4] Department of Preventive Medicine, University of São PauloSão Paulo 05508-270, SP, Brazil
[5] Laboratory of Veterinary Parasitology, Veterinary Teaching Hospital, University of Western São Paulo, São Paulo 190019-70, SP, Brazil
[6] Department of Animal Science, School of Life Sciences, Pontifical Catholic University of Paraná, Curitiba 80230-130, PR, Brazil
[7] Department of Comparative Pathobiology, College of Veterinary Medicine, Purdue University, West Lafayette, IN, 47907, USA
* Correspondence: abiondo@ufpr.br; Tel.: +55-41-3350-5623

Supplementary Materials

Table S1. Univariate analysis for accessing bred, age and hunting behavior as risk factor for anti-*Toxoplasma gondii* antibodies detected by IFAT test in dogs from island and seashore mainland areas of southern Brazil (N = 283).

Variable *	anti-*T. gondii* antibodies		Univariate Analysis	
	Seropositive (%)	Seronegative (%)	OR (95% CI)	*p*-value
	66/283 (23.3)	217/283 (76.7)		
Bred				0.087
Purebred	13 (20.0)	67 (32.1)	Ref.	
Mixed breed	52 (80.0)	142 (67.9	1.87 (0.97–3.81)	
Age				0.005
<1 year	1 (2.0)	34 (18.7)	Ref.	
1 to 8 years	39 (79.6)	132 (72.5)	8.79 (1.81–212)	
>8 years	9 (18.4)	16 (8.9)	16.4 (2.68–433)	
Dog hunting behavior				0.155
No	34 (57.6)	132 (68.8)	Ref.	
Yes	25 (42.4)	60 (31.2)	1.62 (0.88–2.95)	

* Missing data values for breed (9/283 = 3.2%), age (52/283 = 18.4%) and dog hunting behavior (32/283 = 11.3%). The variables were analyzed separately due to the number of missing (age and hunting behavior: lost of information higher than 10%) or imprecision of data regarding bred provided by dog's owners.
